# Raising a Ghost It Were Better to Lay

**Published:** 1917-10-13

**Authors:** 


### The Hospital

The Workers' Newspaper of Administrative Medicine and Institutional Life, Administration, National Insurance and Health.

No. 1636, Vol. LXIII. SATURDAY, OCTOBER 13, 1917. PRICE ONE PENNY

### RAISING A GHOST IT WERE BETTER TO LAY.

The acceptance by the voluntary hospitals of a grant from the War Office in respect of each wounded soldier treated by them may prove a turning-point in institutional history. This was felt at the time, for it was only after a year or so of war that even all the London hospitals brought themselves to accept it. Once accepted, the grant became more than ever a subject for meditation. Was it enough? If not, why not? The first question was easily answered. There were various answers to the second. The desire for an increased grant found, as desires will, many reasons to support it. First, it was pointed out the grant covered, and was intended to cover, the cost of maintenance only, since it was doubtful whether even this expense was met by it, the next step was to decide what other expenses should be placed to its debit. Elsewhere in the present issue we note the suggestion that the grant ought to be added to "on account of capital expenditure." In other words, it is urged by some that the War Office should pay rent and interest on hospital buildings and capital. But the question is more complicated still.

In respect of the grant, another avenue of approach has been found in the case of the disabled soldier. The treatment of these discharged men in civil hospitals has already exercised the British Medical Association. They have been singled out by the Association apparently because the Ministry of Pensions has instructed local pensions committees to pay the hospitals for treatment given to those discharged disabled men who require it. These payments, it is important to note, were intended to cover maintenance only, and range from three to four shillings per man per day in the case of in-patients, and from sixpence to half-a-crown in the case of each visit to an out-patient department. In consequence of the Ministry's action in the matter, the British Medical Association has invited hospital boards to consider the adequacy of these grants, and to share its opinion that the State should pay *also* for professional attendance. The invitation is familiar by this time, and so is the Association's subsequent action in advising hospital medical committees to press hospital managers to adopt the Association's proposal.

The matter must seem rather hard to the Pensions Minister, since for nearly six months he has possessed a medical advisory committee. After some heart-searchings and divided counsels the advisory committee is understood to have agreed that payment for professional services to discharged disabled men at the voluntary hospitals should be demanded on the important condition that the arrangements should be left to a central authority. The point of this condition, apparently, was a desire to avoid embroiling the voluntary hospitals with the local pensions committees in an old, but latent, controversy over the payment of honorary staffs. Since the Ministry of Pensions did not bow to the revised decision of its medical advisory committee, the British Medical Association pressed the medical men on the different hospital staffs to undertake the task of converting hospital committees to its view in the manner above referred to.

It will be evident that the Association has attempted to make hay while the sun shines, and to press its point in respect of the treatment of disabled soldiers, not because the treatment of these men is different in principle from that of undischarged sick and wounded soldiers, but because it has happened that the Ministry of Pensions has issued the instruction for the payment of the latter's cost of maintenance. In fine, the Association appears to think that local Pensions Committees will prove easier to milk than the central authority of the War Office, which, of course, issues the capitation grants in respect of undischarged men. We allude to the matter for two reasons. First, we do not greatly like the Association's tactics, but would prefer a definite policy in favour of the abolition of honorary staffs and the substitution of paid medical staffs everywhere. Secondly, the impression created on the public by the Association's procedure is unfortunate, as can be judged from a careful and succinct article in the *New Age* of September 20, on which we have not hesitated to draw for its convenient marshalling of events. It is true, of course, that, did the British Medical Association announce a policy for the payment of hospital staffs, a big question and an acute controversy would follow. But, in our view, the question itself is so big and important that, if it is to be raised now, it should be raised completely and plainly. Further, the fact that the Association has preferred to side-track the matter proves to us that, though the whole question will doubtless be raised eventually, its present movement is inopportune, and may persuade the public that the profession is eager to grab, which, to say the least, is an exaggeration, though one of which the tactics of the British Medical Association rarely offer evidence in disproof.

